# Developing Digital Games to Address Airway Clearance Therapy in Children With Cystic Fibrosis: Participatory Design Process

**DOI:** 10.2196/games.8964

**Published:** 2018-11-21

**Authors:** Fabio Balli

**Affiliations:** 1 Breathing Games Montreal, QC Canada; 2 Concordia University Montreal, QC Canada

**Keywords:** digital games, children, cystic fibrosis, game design, treatment adherence and compliance, intersectoral collaboration, access to information, recommendations, telemedicine, mobile phone

## Abstract

**Background:**

Children affected with cystic fibrosis do respiratory exercises to release the mucus stuck in their lungs.

**Objective:**

The objective of our study was to develop prototypes of digital games that use breath pressure to make this daily physiotherapy more fun.

**Methods:**

We used a participatory design approach and organized short events to invite contributors from different disciplines to develop game prototypes. From the 6 prototypes, 3 were tested by 10 children during a prestudy. The source code of the games, of which 2 continue to be developed, has been released on the internet under fair use licenses.

**Results:**

We discuss 7 themes of importance in designing games for health, combining our experience with a review a posteriori of literature.

**Conclusions:**

This study provides examples of games and their pitfalls as well as recommendations to create games for health in a participatory approach that enables everyone to improve and adapt the work done.

## Introduction

### Burden and Treatment of Cystic Fibrosis

Cystic fibrosis (CF) is a chronic disease estimated to affect 70,000-100,000 people worldwide [1]. Although advances in therapeutic care may now provide a life expectancy of >40 years [2], respiratory diseases still represent the main cause of morbidity and mortality [3]. The expectoration of airways’ surface bacteria is normally assured by a layer of slippery mucus produced by the lungs’ epithelial cells. Because the mucus is thick and viscous in patients with CF, mucus and bacteria accumulate, which results in the obstruction, infection, and inflammation of the airways. Therefore, reducing the progression of respiratory diseases is of the utmost importance to prevent early death in patients with CF [4].

Airway clearance techniques, such as positive expiratory pressure (PEP) physiotherapy, have been highlighted as effective means to diminish an airway’s obstruction, infection, inflammation [5], and subsequently reduce pulmonary exacerbation [6]. By expiring through a tube that limits the flow of air, the device forces to make longer expirations, which help in keeping the airways open and removing the mucus stuck in the airways [7]. However, the burden of the treatment regimen with regard to CF has been highlighted numerous times [8], especially for children [9]. PEP physiotherapy presents the most time-consuming activity in the treatment, and poor adherence to this therapy is reported in almost half of the children affected with CF [10,11].

### The Use of Digital Games to Increase the Treatment Adherence

Transforming the respiratory therapy into a digital game has shown promising results in increasing adherence [12,13]. Digital games are artificial systems in which players interact, which comprise conflict, rules, and quantifiable outcomes [14]. Games for health is an emerging field of “serious gaming,” that is, playing with a purpose beyond entertainment [15]. Games for health have shown promising results in increasing knowledge, delivering persuasive messages, modifying behaviors, and improving health outcomes [16,17]. Advantages of such games over other therapeutic methods appear in our practice, including the following: making respiratory therapy fun through storytelling and interaction; altering time perception through immersive experience; making the long-term impact of daily therapy visible; developing and validating knowledge through learning sequences and game levels; fostering a constructive mindset through meaningful quests and collaborative gameplay; and collecting data on a regular basis about the effectiveness of the therapy.

However, Baranowski et al [16] emphasized that games for health should be much more engaging than the ones currently available to be truly effective. They recommend involving multidisciplinary stakeholders in all development phases to ensure the usability, desirability, and feasibility of games. Involving children affected could help them take ownership of their care by designing levels and gameplays to make their treatment fun.

This paper presents an overview of the participatory design process [18] used in the development of 6 games aimed at increasing the adherence to PEP therapy. The games were based on the traditional PEP therapy technique, in which an electronic pressure sensor was added to the PEP mouthpiece, transforming breath to a digital signal used to control the game character. In addition, this study will briefly describe each game that was developed and present the pilot evaluation we used. Because the development process of games for health and discussion of failures in their design are rarely documented [19], we will conclude by summarizing the challenges we faced and by stating what we learned, so that it might help researchers and practitioners avoid the same pitfalls.

## Methods

### Participatory Design Process

**Figure 1:**
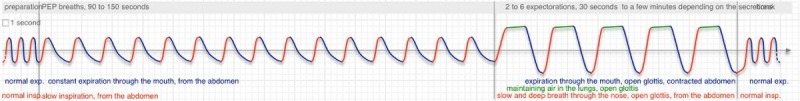
A graph of positive expiratory pressure exercise, own representation. Red lines, inspirations; green lines, holding; blue lines, expirations. Credit: breathinggames.net.

**Figure 2:**
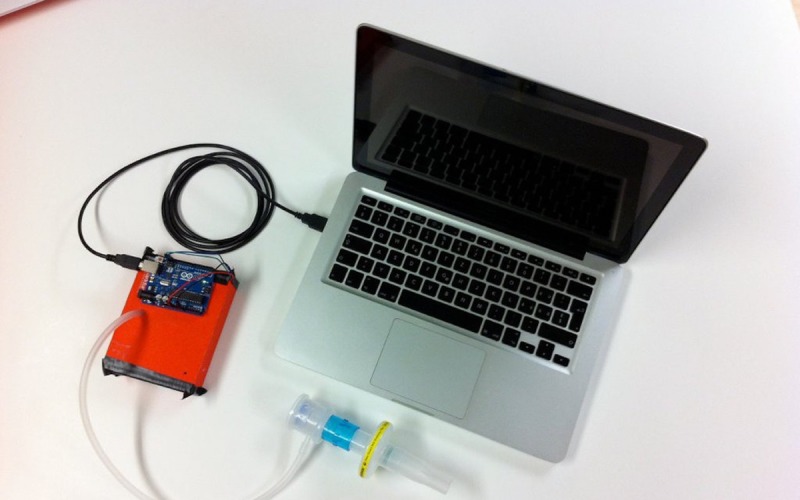
A device transforming expiratory breath into digital data through a pressure sensor. Credit: breathinggames.net.

The project started in January 2014 between 2 game design students and the CF pediatric team at Sainte-Justine Hospital (Montreal, Canada). The clinicians were looking for a novel way to make respiratory therapy more engaging for their young patients. An initial environment scan and literature review targeting CF games showed us only a few games (*AstroPEP*, *CFpal*, *Creep Frounter*, *Flower for all*, *KIbreath*, *LudiCross*, and *MyCarnival* [12,20]), which were all very basic games and were not available to the public. Discussions enabled the students to understand the PEP exercise to build the game (Figure 1). Furthermore, an electronic pressure sensor that was developed previously was used so that breath could act as an input for the game (Figure 2) [21]. The project was then named “Breathing Games” [22].

### Initial Developments

**Figure 3:**
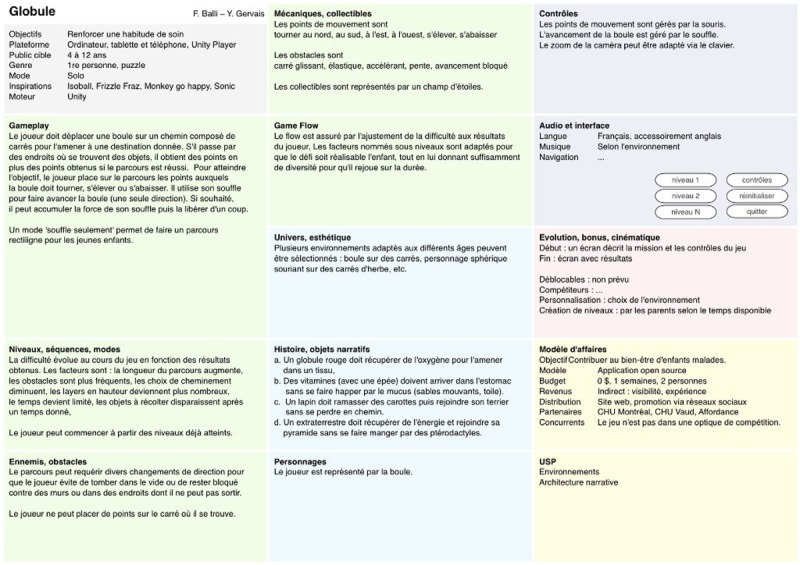
The document describing the key elements of the game Globule. Credit: breathinggames.net.

It took up about 800 hours to develop the 3 initial prototypes—*Globule*, *Ange-Gardien*, and *PEP Hero*. The key elements of the games were defined (Figure 3), including goals, gameplay, levels, obstacles, mechanics, game flow, visual universe, story, and character. The aim was to experiment with different game mechanics to test with children. *Globule* was designed as a simple puzzle, which could easily include a level editor so that children could create their own levels. *Ange-Gardien* was conceived as a tower defense, where children could manage different resources with an increasing level of difficulty. *Celebrations* was designed as a contemplative game, which inspired from open worlds, where children could explore different spaces without a defined goal. In *Globule*, the breath exercise served to collect elements that could be used in the game; in *Ange-Gardien*, breath activated towers that would kill enemies; in *Celebrations*, it gave energy to the character so that it may move. *Globule* and *Ange-Gardien* were developed part time between January and April 2014 by 2 game design and 2 sound design students. *Celebrations* was abandoned because of the resources required to develop a multiplayer game.

In February 2014, the team took part in a 3-day cocreation event (hackathon) organized at Sainte-Justine. The team presented the project, and 5 young professionals with skills in visual arts, user experience, electronics, and communications joined. The team then created *PEP Hero*, a side-scrolling game. One participant worked on the user interface, another on the visuals, 3 on the design and coding, 2 on communications, and one on the electronics.

**Figure 4:**
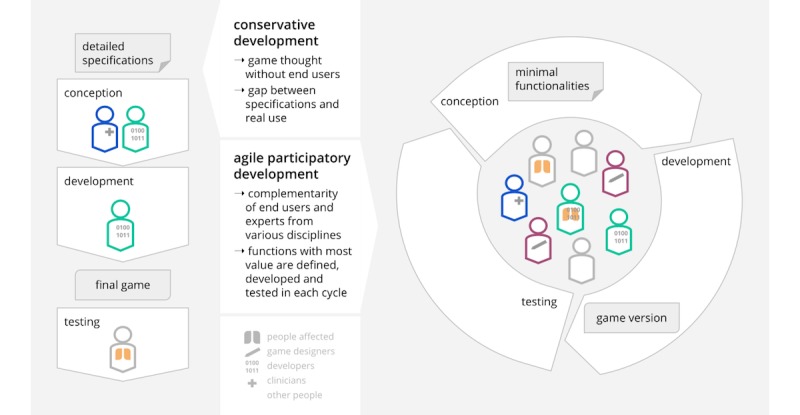
Agile participatory development of games. Credit: breathinggames.net.

Our approach to developing the games was pragmatic; we designed the games and the CF team was available for advice. We developed the prototypes in cycles according to the value created for the end user (agile method, Figure 4). All contributions were made without funding. We used the broadly used game engine Unity 3D, which, unfortunately, is not free (libre) and open source.

Beyond the iterative process, people from different disciplines, including game designers, software developers, visual artists, sound composers, electronics engineers, physiotherapists, and pulmonologists, were involved. Children affected were involved in testing, but we were not able to mobilize patients with CF and family community during the cocreation process. We hypothesize that this is because of the burden already related to CF care. Involving end users is however important to develop more effective games [17]. A critical reflection on the role of serious games was also done during this period [23].

### Initial Tests

To test the prototypes, parents of children who had to come to the CF clinic for a visit were contacted by phone; those interested were invited to meet the game designers during their visit. Written consent was obtained from the 10 participants. Children aged 7-11 years answered a preliminary questionnaire, which focused on their play preferences (eg, favorite game, time spent playing daily) and design preferences (eg, favorite music to relax to, favorite designs for a spaceship). Children were then invited to play 2 or 3 games, each game having 1 or 2 levels. Each game was presented before they played. Children then answered the second questionnaire to evaluate each game (eg, difficulty of the game, integration of physiotherapy, what should be improved, favorite and worst moment in the game). The whole process required about 30 minutes with about 5 minutes of play. The prestudy was approved by the Sainte-Justine ethics panel (#3918).

In the first questionnaire, children answered questions on their access to various technologies—internet, laptop, computer, tablet, smartphone, and the Microsoft Kinect (often used in active video games on CF). They had started their treatment between ages 7 and 9 years and underwent it alone, accompanied, or both. When they did not want to do the treatment, they answered that it was because they had no time, they did not think about it, or because it was boring; 2 children said that they always did their therapy. They wanted to do the treatment for their health and their parents. In addition, children reported they played games for 5-60 minutes per day. Questions about preferences—favorite game, movie, book, hero, color, animal, relaxing music, the game they play the most, and the activity they like—resulted in very different answers. We had planned that such questions would help us customize the games. However, the children needed sample answers, and their proposals were often too diverse to inform us.

### New Developments

**Figure 5:**
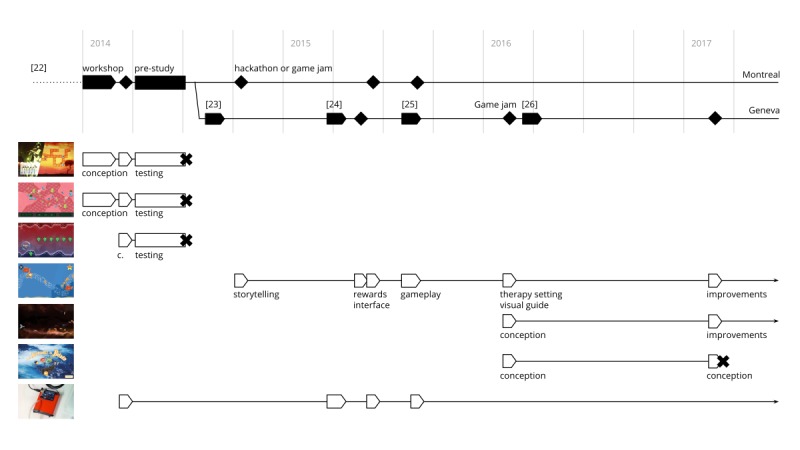
Timeline of the game development. Credit: breathinggames.net.

It took over 1000 hours to develop 3 further prototype games: *Heritage*, *Bloïd*, and *Les aventures du Briand*. Building on the previous learning, we conceptualized *Heritage* in November 2014. The game was developed during 5 cocreation events by >20 contributors. In addition, 3 students in software development worked respectively on the breath input [24], the game design [25], and the creation of a game core [26]. *Heritage* included a settings panel so that the exercise could be adapted to practices of different countries. *Bloïd* and *Les aventures du Briand* were conceived during a game jam in March 2016 and continued at another game jam in February 2017 (Figure 5). These 3-day cocreation events were called jams to appear less technical and involved 15 contributors and collaborations with the University Hospital of Canton Vaud (CHUV), the Genevan Federation against CF (FGLM), and Lift Conference. In addition, a participatory design process similar to the one adopted for the prototypes was used. In these 3 games, the breathing exercise is fully integrated to the gameplay, and the breathing cycle is not represented as a chart.

### New Tests

*Heritage* and *Bloïd* were tested informally during the game jams by a few children and adults, who gave a positive feedback. A mixed-methods study is being prepared to test these games. *Les aventures du Briand* requires further development before it can be tested.

## Results

### Games Developed

#### Globule

##### Gameplay

**Figure 6:**
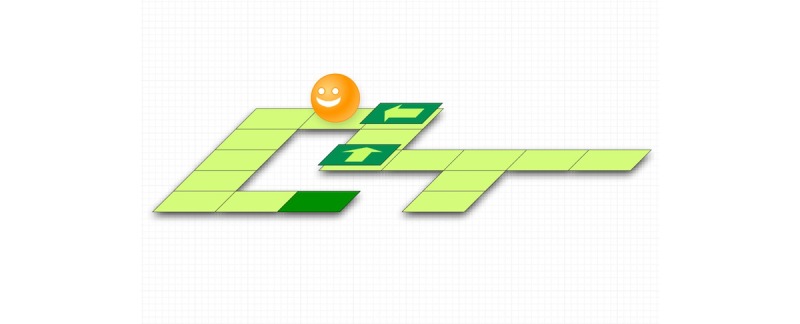
The sketch of the game Globule. Credit: breathinggames.net.

**Figure 7:**
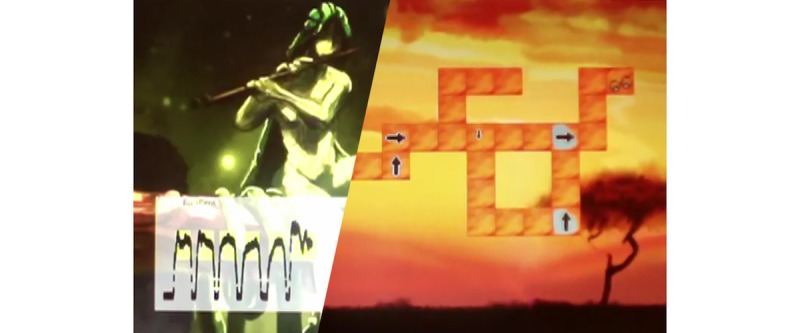
The visual of the game Globule: breathing exercise to collect arrows (left), and puzzle (right). Credit: breathinggames.net / F Moncomble.

*Globule* (Figures 6 and 7) is a puzzle game that uses an expiratory pressure device. The breathing exercise and the gameplay are separated to potentially add the breathing component to existing games. Each game level is composed of the following 3 phases: 1) children do their breathing exercise based on the therapeutic pattern; for each breath done with the right duration and pressure, children receive an arrow pointing north, south, east, or west; 2) children have a break to expectorate mucus; and 3) they play the puzzle by placing the arrows to direct the character toward a goal. Children are also able to select among 3 environments: the desert, the jungle, a futuristic universe.

##### Tests

Overall, 8 children tested the prototype [27]. After playing, most children found that the game was very fun, how to play was easy to remember, doing the therapy while playing was easy, and that the game was adapted to the treatment. All children liked to play with the arrows, path, and the level that was more difficult. One child did not understand the purpose of the game.

Regarding improvements, children proposed to add characters, have more time to set the arrows, add guitar music, correct the mistakes, correct the exhalation time, and connect it to the hardware (we had a problem with the pressure sensor in that individual test). In addition, children suggested we add levels, music, and more variety to make the game interesting over many weeks. Children mostly rated the game high; the first child testing the game gave a negative evaluation because the pace of the exercise was too fast, which was corrected.

##### Strengths

Based on our observations, children were able to perform the expiratory time and constant pressure required for the therapy, and get rewarded for their performance. The puzzles are easy to customize and construct (visuals for the squares, protagonist, and background), which could enable children to create and share their own maps. The minimalist story (collecting arrows to reach a goal) seemed to positively impact children.

##### Limits

The puzzle in itself has no connection to breathing. Parents mentioned that separating the breathing exercise and the game requires more time than the therapy alone, which they see as inadequate, given the time already required daily.

##### Status

The development of the game was discontinued due to the aforementioned limits.

#### Ange-Gardien

##### Gameplay

**Figure 8:**
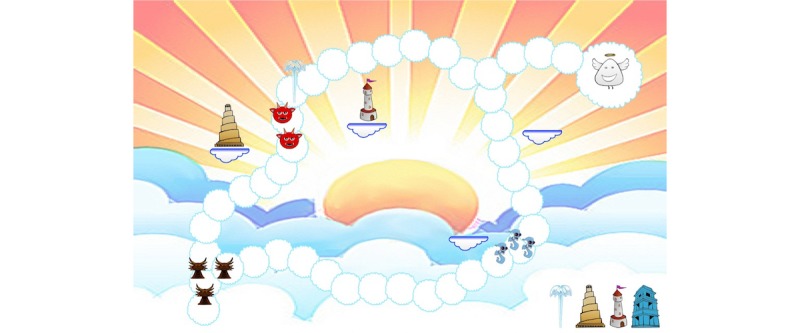
A sketch of the game Ange-Gardien. Credit: breathinggames.net.

**Figure 9:**
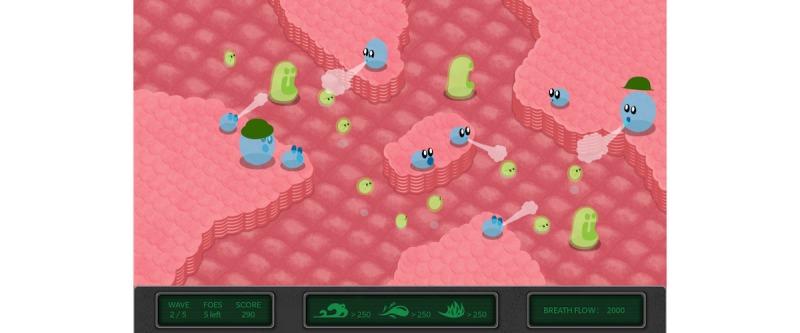
Visuals for the game Ange-Gardien. Credit: breathinggames.net / J Danger.

*Ange-Gardien* (Figures 8 and 9) is a strategy tower defense game that uses an expiratory pressure device. Here, the breathing exercise and the game are combined. Children defend the little guardian angel that fell from his shoulder after being kicked by the devil on the other shoulder. The devil wants to capture the angel before the child saves it. When the game starts, the child sees a map with different paths and places to build towers. The child receives a certain number of points to build different kinds of towers, representing wind, fire, and water. Then, enemies arrive on different paths. The child clicks the tower to activate, and breathes out to make that tower shoot at the enemies around. Each devil destroyed gives points. Each demon reaching the other side of the map reduces the number of lives of the angel. After 5 seconds, the towers deactivate, prompting the child to inspire. When the breathing cycle is done, no more enemies appear because children must do their expectorations. On the next level, towers can be built or upgraded using points. The game was developed and tested on the basis of geometric shapes; visuals were prepared but not integrated into the game.

##### Tests

Only 2 children tested the game because of delays in the development. After playing, children found that the game was very fun, remembering how to play was easy, doing the therapy while playing was easy, and that the game was adapted to the treatment.

Children liked to set the towers and the evolution of the scene. Regarding improvements, a child proposed to correct a bug in the gameplay. In addition, children suggested adding levels, colors and other visuals, towers, enemies to make the game interesting over many weeks. Children rated the game high on a scale from 10 (best) to 1 (worst).

##### Strengths

The mechanics can evolve from very simple to complex strategic gameplay and breathing cycles can easily be related to different parts of the gameplay. The game can be adapted to multiple players and can also include input from the children’s relatives.

##### Limits

Although this was not brought up by the testers, the angel devil may not be well perceived depending on religious beliefs. In one design, the enemies are represented as mucus; this created a discussion whether integrating elements of the disease in the game would support or discourage the children in their efforts.

##### Status

The development was discontinued because of major changes in the game engine. The gameplay should however be reused.

#### PEP Hero

##### Gameplay

**Figure 10:**
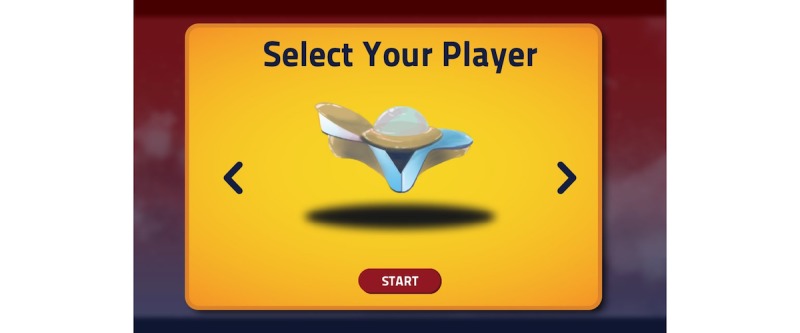
A visual of the game PEP Hero. Credit: breathinggames.net / K Berthiaume.

**Figure 11:**
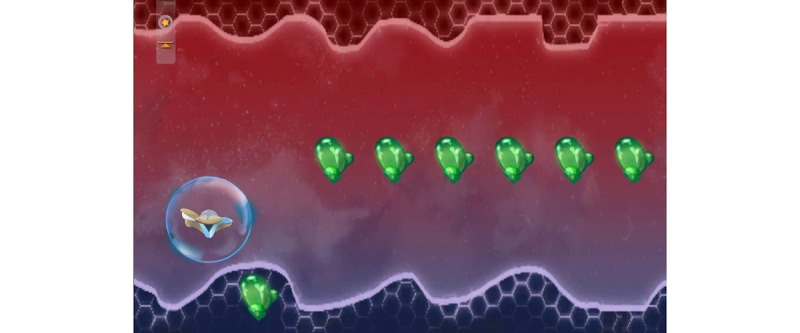
A visual universe of the game PEP Hero. Credit: breathinggames.net / K Berthiaume.

*PEP Hero* (Figures 10 and 11) is a side-scrolling space shooter that uses an expiratory pressure device. The game simply adds a visual universe on the top of the therapeutic exercise. The child is the captain of a spaceship that goes up when breathing out and down when breathing in. By directing the vessel, the child is able to collect items. Afterwards, the child has a break to expectorate and can then start a new cycle. The game included various vessels and items to collect, but their selection was not functional during the tests.

In the first individual test, the green items were spread over the screen; this did not fit with the exercise because the child did not know when to breathe. The items were then aligned, which helped the child to have a continuing exhalation but did not show them when to breathe in. Thus, additional items were added for the inspiration phase, that helped the child adopt a regular breathing pace.

##### Tests

Overall, 8 children tested the game. After playing, most children found that the game was very fun, remembering how to play was easy, doing the therapy while playing was easy, and that the game was very adapted to the treatment.

Children liked everything, including the ending, the visuals, and the first level. Regarding improvements, children proposed to increase the duration of inhalations (corrected after the individual test) to make the first expected breathing visible and solve a bug. Children suggested to add other elements such as levels and colors to make the game interesting over many weeks. Children rated the game high on a scale from 10 (best) to 1 (worst).

##### Strengths

The strength of the study was that simple mechanics mimicked the breathing exercise with the advantage of giving direct feedback about the correct pressure and duration of breathing. Visuals can be easily adapted.

##### Limits

A long-term interest could be hard to ensure because the mechanics are very simple.

##### Status

The development was discontinued, as *Heritage* was created, developing further similar mechanics.

#### Heritage

##### Gameplay

**Figure 12:**
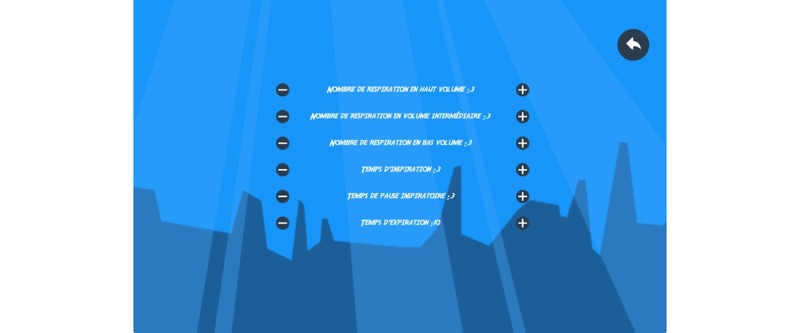
A visual universe of the game Heritage: the setting of the exercise. Credit: breathinggames.net / N Wenk.

**Figure 13:**
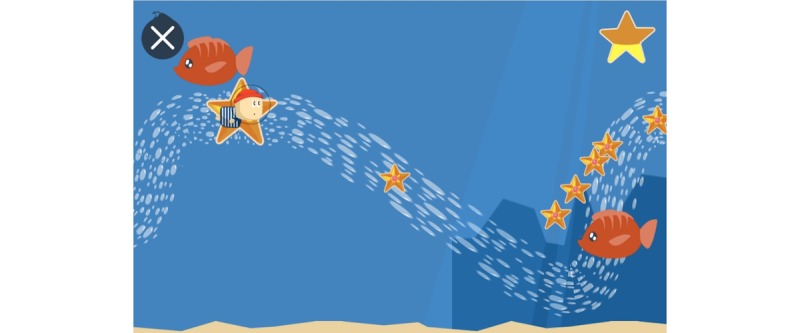
A visual universe of the game Heritage: breathing exercise. Credit: breathinggames.net / C Skrapits / N Wenk / J Danger.

*Heritage* (Figures 12 and 13) is a side-scrolling game using an expiratory pressure device, in which a child defends the cultures of different countries. For Greece, for example, the character has to catch a thief who has seized Poseidon’s trident-fork. If he recovers it, the character must then find who stole Aphrodite’s potion, Zeus’ lightning, Gaia’s seed bag, Hermes’ shoes, and Athena’s rouet to collect more points. In the end, the God of the Seas will then prepare the meal for the party with his trident-fork; Aphrodite will take care of the atmosphere; etc. During the journey, a child learns facts and figures about different countries.

A table was created during a cocreation event to imagine different stages (daily exercise) with each stage having 6 levels (breathing cycles). For each level, cultural elements were defined. Peru, for example, was proposed by a participant who knew the country. For Peru, the background elements would be the sky, nonplayer supporting character would be the goddess *Pachamama*, the stolen object would be the earth, the transport means would be a condor, obstacles would be clouds, the asset would be an *unku* (piece of cloth). Graphic elements were created by different artists, who, while keeping the same line, always changed parts of already existing elements.

#### Bloïd

##### Gameplay

**Figure 14:**
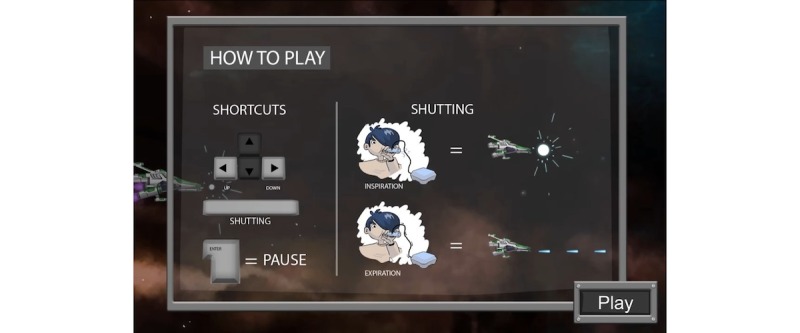
A visual of the game Bloïd: introductory explanations. Credit: breathinggames.net / A D-Pierson / K Piccand.

**Figure 15:**
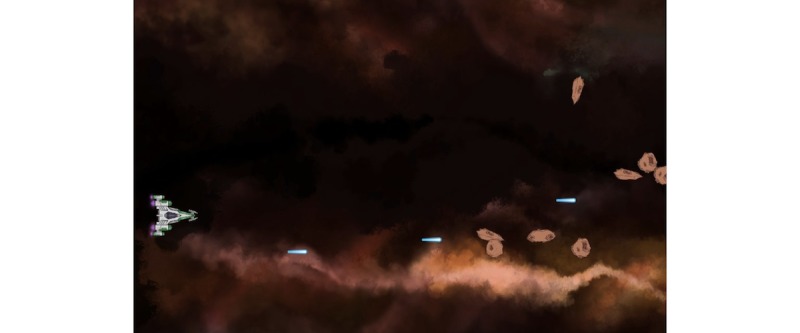
A visual of the game Bloïd: breathing exercise. Credit: breathinggames.net / A D-Pierson / K Piccand.

*Bloïd* (Figures 14 and 15) is a side-scrolling space shooter that uses an expiratory pressure device. In this game, a spaceship has to thwart the traps of an intergalactic journey to reach a mysterious planet mentioned in a prophecy. When the child breathes in, meteorites appear in front of the spaceship; when the child breathes out, projectiles are launched to destroy the meteorites. The keyboard is used to direct the ship. Over time, the number of meteorites that appear at each inspiration grows to encourage players to extend their expiration, slow down their pace, and develop their lung capacity; this enables them to gain a better score. The visuals were created to be realistic and favor immersive play.

Initially, the goal was to create a game similar to the previous ones based on the traditional CF exercise. The team of designers, developers, musicians, and patients found however that the proposed pattern was too limiting to create compelling gameplay that would really increase adhesion. Hence, they decided to create a more flexible exercise, in which breathing would be less constrained. *A posteriori*, the gameplay relates more to a heart rate variability exercise (cardiac coherence) that can help cope with stress.

#### Les Aventures du Briand

##### Gameplay

**Figure 16:**
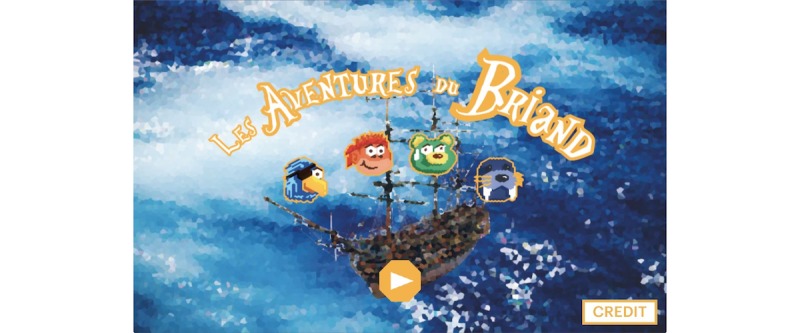
A visual of the game Les aventures du Briand: start screen. Credit: breathinggames.net / J Danger.

**Figure 17:**
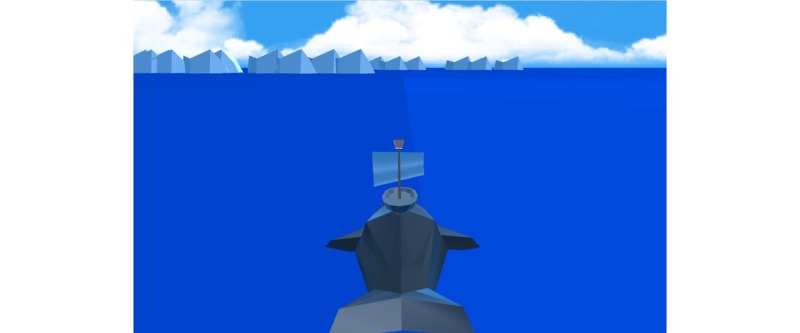
A visual of the game Bloïd: breathing challenge. Credit: breathinggames.net / J Danger / C Koker.

*Les aventures du Briand* (Figures 16 and 17) is an adventure game that uses an expiratory pressure device. In this game, a player is the captain of a ship, who has to breathe to solve challenges faced by the crew. The game is composed of different mini-challenges related to breathing exercises. Similar to *Bloïd*, the team that conceived the game wanted to break free from a strict breathing exercise and instead, opt for a more flexible pattern. The outcomes of the game need to be further researched on.

## Discussion

### Principal Findings

Our approach to developing these games was pragmatic; we applied our understanding of game design to achieve a goal defined in collaboration with clinicians. Although we collectively discussed and documented key elements of different games, we did not follow any specific methodology but built on our experience and intuition. In this section, we synthesize findings from publications collected around digital games conception. Building on this new body of knowledge, we highlight key elements and learnings in the field that we had realized beforehand. The 7 themes presented are based on recurring elements mentioned in the publications selected.

### People Involved in the Creation

#### Literature Findings

Games for health are highly influenced by the team conceiving them—the diversity of backgrounds, their perspective of health (from the reductionist biomedical management of a disease to a holistic promotion of well-being), and the level of participation in decision making.

End users should be actively involved in the creation process—their input about their pleasure of playing, interests, ways of learning, and beliefs can help make the games more relevant [28]. Multidisciplinary teams can help them translate their ideas into design elements [29]. Experts, on the other hand, can help integrate health knowledge and broaden the narrative [30]. Medical care professionals can ease the interactions with the patients while keeping ethics in mind [31] but will often not be able to evaluate the game or its safety [32].

DeSmet et al [17] shared a participatory approach that seems more adapted to create narratives, characters, and other game elements than to develop gameplay.

#### Learning Outcomes From the Field

The involvement of young professionals in game design, software development, visual arts, and project management was essential to realize the prototypes. In addition, electronics engineers, communications specialists, and people in the free software community contributed to building the breathing sensor, increase our Web-based outreach, and sketch the sociolegal framework, respectively.

Despite collaborations with patient associations, we were able to mobilize only one CF family through all of our events. To address this gap, patients with CF have been recruited by our partner hospital for an upcoming event in France. The contribution of medical care professionals was particularly useful in setting the goals, mechanics, and content for the games, and to answer questions during the events. Owing to their status, they may, however, overshadow the experience and needs of patients and push the focus on managing the disease versus the promotion of health and well-being of a person overall in the long term. Bringing too much medical content may reduce the fun and impact of a game.

### Cocreation Process

#### Literature Findings

When creating a game for health, designers can either copy a successful example of gameplay, integrating the medical content into the story, or conceive a game that embeds medical information into the gameplay. The first can help in memorizing information, whereas the second allows for targeting more complex learning needs [15].

Working in short cycles gives the ability to attain better results, value rapid learning, and fine-tune the existing work in a short period. Dow et al [33] stated that contributors working in cycles on a new task are as efficient as contributors working sequentially on a task they master.

#### Learning Outcomes From the Field

Given that the work evolved through events, the games were developed using an agile methodology: prototypes were designed and a minimal viable product was developed and further improved by prioritizing the functions to be added. Because the aim was to make a well-defined physiotherapeutic exercise more attractive, the team worked on how to create gameplay around it. In a less binding context, creating a story and gameplays where health-related content can be easily embedded appears more appealing.

The immediate documentation of the work, including uploading the source code of the game, and of editable visual and sound elements is essential. The tools and the fair use licenses used are described in another paper [18].

One key learning outcome after hosting many health game jams is that such events are well adapted to raise awareness, foster collaboration, recruit long-term contributors, and create a multitude of prototypes to test different ideas. Because they are ephemeral, these events, however, do not allow the creation of high-quality games. Prototypes realized during game jams can, however, be redeveloped by a multidisciplinary team of professionals and tested during dedicated cocreation events.

### Gameplay

#### Literature Findings

Sciart described gameplay as “a ludic experience regulated by game rules, mediated by game mechanics, and oriented to the satisfactory achievement of goals predetermined by the game and agreed upon by players.” page 103 [34]. Gameplay can be separated into the rules of the game world (mechanics), a player’s interaction with these rules (dynamics), and the emotional experience that emerges (affects) [15].

Orji et al [35] propose to either create a variety of gameplay (“competition and comparison, cooperation, customization, personalization, praise, self-monitoring and suggestion, simulation, and reward,” page 473) that can mobilize different kinds of players (“achiever, conqueror, daredevil, mastermind, seeker, socializer, and survivor,” page 473) or adopt the gameplay that better answers all players, possibly through comparison or self-monitoring. Indeed, gameplay can fill different kinds of needs—one player will like to explore, the other to compete, the third to socialize; one will like fast actions, another to solve problems, and a third one to build strategies. To help design the player experience, the following 6 elements can be checked: voluntary participation; a commitment toward a goal; a physical involvement; the creation of meaning; the perception of feelings; and the creation of social relations [36].

#### Learning Outcomes From the Field

The types of games (quiz, adventure, shooter, tower defense, etc) were considered for the prototypes rather than categories of players or experiences. Given the time required for the therapy, the games that separated the respiratory exercise from the gameplay were abandoned. Even basic gameplay and simple stories were positively perceived by children. For each game project, the teams focused on developing one level of the game with a polished visual, sometimes sounds and music, and an end screen. This, however, does not allow us to test the evolution of the gameplay and challenges throughout the games.

For future events, it appears more adequate to conceive many levels with placeholders (eg, colored shapes instead of designed elements) and to develop in parallel a database of visual and sound elements that can be used in the different levels or games.

### Storytelling

#### Literature Findings

In games for health, narratives can help players develop skills, experiment with new situations and behaviors, and change their beliefs in regard to health. For children, fantasy and nonsensical elements can increase their involvement and fun and encourage them to develop their creativity [37]. Death and other sensitive subjects can be included when learning is provided [38]. Addressing situations that affect players and creating meaningful decisions allow them to reflect on their ideals and values and possibly learn about societal issues [38]. In addition, the game creators have to decide the extent to which the story can differ from the mainstream knowledge about health.

Sciart [34] recommends that the game evolves according to the not-too-obvious decisions players have to make, and that players should not be able to replay and change these decisions. Khaled et al [39], however, states that players often expect to replay to improve their experience. The number of choices should increase during the game as players strengthen their skills, and this also increases the game effectiveness [17].

#### Learning Outcomes From the Field

Although a story, and sometimes a sketchboard, was written for different games, it was only appearing through the visuals and not in the initial gameplay. While this can easily be added afterwards, for example, by cropping existing characters and creating a dialogue between them, it did not allow us to really test and improve the storytelling. For *Heritage*, the story was built for different levels, each building on a different culture. For other games, the story generally gave some context and the quest. A reflection on what meaningful decisions players can take would certainly improve the quality of these narratives. Furthermore, games could include time references (day or night, seasons, etc) to diversify the experience. An unsolved question is the impact of including elements related to CF (lung, mucus, etc) in the game.

### Character and Customization

#### Literature Findings

For Khaled et al [39], developing the relation between players and their character impacts the immersion and appropriation of knowledge beyond the game. Being able to customize the character can help, even if it does not add much to the gameplay. The interaction sought by the designer should define a player’s perspective; should they have a global vision of the world, should they be embodied, or both? Using nonhuman androgynous characters or fully customizable characters can help avoid stereotypes often seen in mainstream games. Guidelines to avoid marginalization, for example, providing an option to zoom, are available [40].

#### Learning Outcomes From the Field

The characters developed were nonstereotyped and included animals, aliens, angels, a starship, and some people. For *Heritage*, one idea was that players could collect and wear clothes from different cultures. No customization was implemented because of a lack of resources. Inviting visual artists to create a database of elements to customize the characters could be valuable.

### Feedback and Rewards

#### Literature Findings

Players need not focus on how to play but to immerse themselves in the experience. Learning how to play should be easy and should not require a tutorial or explanation. Sufficient feedback should be provided but not so much that the players get distracted [40]. Health information should be of real use for patients, and additional information should be available on demand. Visual representations and colors should be valued [41]. Rewarding players with points, material, and other awards is useful as long as it creates meaning for players. Intrinsic motivation should be sought and encourage the feelings of autonomy, competence, and belonging. Feedback may be adapted to the social context of players too [42]. For Flanagan and Nissenbaum [43], the reward system explicitly highlights the values behind the game; it can value creativity, competition, exploration, social interactions, etc. For them, players should choose whether they want to compete.

#### Learning Outcomes From the Field

The visual representation of the breathing exercise was central in different games. Players received items when doing the exercise correctly, and a gauge showing the on-time breath was also visible in some games. Although the representation helps players know what is expected, it also limits the interest in the long term. *Bloïd* hid the exercise behind its gameplay—the breath in brought meteorites, breathing out allowed the player to shoot at them, and the number of meteorites grew over time to encourage a slow breath; this allowed us to create more immersion and shift the focus away from the medical element. *Les aventures du Briand* intended to have many mini-games whose results would impact the global quest.

For all games, the rewards were closely linked to the breathing exercise and were only positive: the team did not want to penalize a wrong exercise but encourage a good one. There was a discussion about creating 2 separate reward systems, one for the health-related part and another for the quest; however, this was not implemented. Better integrating these questions while defining the story and gameplay could help ensure more variety and meaning in the rewards.

### Evaluation and Diffusion

#### Literature Findings

Games for health should be validated with research and also be easily accessible and foster reliable information, technical efficiency, data privacy, and ease of use. The regulation regarding medical devices is changing. The French Health Authority published best practices covering these issues [44] and the Food and Drug Administration is reviewing its process [45]. Health Canada recommends checking if regulation exists, especially when the software influences decisions on health [46]. Beyond best practices, a “Mobile Application Rating Scale” was developed by the Queensland University [47]. Graafland et al [48] created a specific tool to assess the didactic and interactive experience of games for health.

#### Learning Outcomes From the Field

Access to health innovation is essential. From the beginning of the initiative, the source code of the games was released online under a license that enables every interested person to use, reproduce, and improve them. In addition, efforts were made to document the cocreation process. As mentioned earlier, the games presented were not tested extensively, but a study is planned to evaluate *Heritage*, *Bloïd*, as well as other games to be developed around CF.

### Conclusions

The participatory design of games for health is an excellent tool to bring together people and introduce them to multidisciplinary work. Recurring short events around the same topic enable participants to prototype and test a large variety of gameplay modes, get acquainted with and raise awareness on health issues, and find contributors ready to engage in the long term. Such events are, however, not adapted to build high-quality games.

From the 6 games prototyped, 3 were abandoned because of unsatisfactory choices in their design (separation between the breathing exercise and the game in *Globule*), technical issues (major changes in the game engine for *Ange-Gardien*), and a scope that was too ambitious (the team working on *Les aventures du Briand* spread before a functional version of the game with mini-games was ready). *PEP Hero* was rewritten as *Heritage* to allow the customization of the breathing exercises. *Bloïd* was improved with additional visuals. Both *Heritage* and *Bloïd* are going to be tested and further developed.

**Figure 18:**
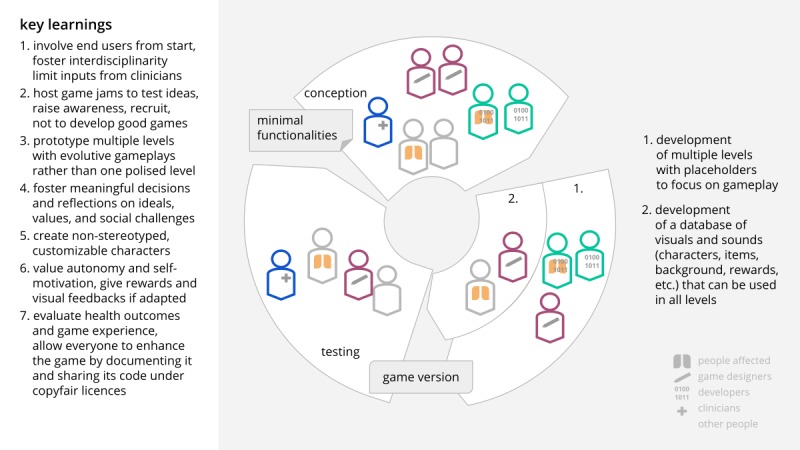
Recommendations to design games for health. Credit: breathinggames.net.

To create further games for health, the key learning outcomes (Figure 18) from the 7 themes addressed are to develop prototypes that show the gameplay for different game levels and to separately create a database of visual and sound assets that can be used throughout the game (customizable characters, universe, rewards, etc). Moreover, the story should encourage meaningful decision making, embed health information while not building the game around it, and foster autonomy and inclusion. As briefly mentioned, special attention should also be given to the evaluation and accessibility of the game, its source code, and its cocreation process so that a maximum number of persons can use and modify it.

## Abbreviations

CF: cystic fibrosis
PEP: positive expiratory pressure
